# Risk Factors of External Ventricular Drain Infection: Proposing a Model for Future Studies

**DOI:** 10.3389/fneur.2019.00226

**Published:** 2019-03-15

**Authors:** Abayomi Sorinola, Andras Buki, Janos Sandor, Endre Czeiter

**Affiliations:** ^1^Department of Neurosurgery, Medical School, University of Pécs, Pécs, Hungary; ^2^János Szentágothai Research Centre, University of Pécs, Pécs, Hungary; ^3^Department of Bio-statistics and Epidemiology, Faculty of Public Health, University of Debrecen, Debrecen, Hungary; ^4^MTA PTE Clinical Neuroscience MR Research Group, University of Pécs, Pécs, Hungary

**Keywords:** (EVD) external ventricular drain, EVD infection, ventricular catheter (VC), ventricular catheter infection, risk factors

## Abstract

**Background:** External ventricular drain (EVD) has a major role in the management and monitoring of intracranial pressure (ICP) and its major complication is EVD infection. The risk factors for EVD infection are still a major topic of controversy, hence the need for further research.

**Objective:** The objective of this review was to identify risk factors that affect the incidence of EVD infection and create a model, which can be used in future studies in order to contribute to elaborations on guideline for EVD.

**Methods:** A PubMed and Google Scholar literature search was performed and data were extracted from studies published from 1966 through 2017. The search of the databases generated 604 articles and 28 articles of these were found to be relevant. A manual search of the 28 relevant papers generated 4 new articles. Of the 32 relevant articles, 20 articles that performed a multivariate analysis of the suspected risk factors of EVD infection and had a positive culture as a mandatory component in diagnosis were selected for data collection and analysis.

**Results:** Because reviewed papers investigated only a few influencing factors, and could not determine convincingly the real risk factors of EVD infection and their real strengths. A total of 15 supposed influencing factors which includes: age, age & sex interactions, coinfection, catheter insertion outside the hospital, catheter type, CSF leakage, CSF sampling frequency, diagnosis, duration of catheterization, ICP > 20 mmHg, irrigation, multiple catheter, neurosurgical operation, reduced CSF glucose at catheter insertion and sex were identified.

**Conclusion:** This review summarizes a set of variables which have to be covered by future clinical epidemiological investigations in order to describe the etiological background of EVD infection.

## Introduction

Traumatic brain injury (TBI) is a major cause of death and disability in the world harboring significant public health and socio-economic importance. TBI is estimated to be the primary cause of mortality and disability among young individuals. In 2013, in the United States, TBI was a diagnosis in more than 2.5 million emergency department visits and 282,000 hospitalizations. According to the Center for Disease Control and Prevention (CDC), TBI contributes to 30% of all injury-related deaths in the United States (1). Epidemiological data on TBI from the European Union are scarce, but do indicate an incidence of hospitalized TBI of approximately 235/100,000/year, although substantial variation exists between European countries (2). TBI is associated with a cost of over $76 billion in the USA (3) and least €33 billion in Europe (4) and severe TBI accounts for 90% total medical cost of TBI (1).

Ventriculostomy is frequently used in the management and monitoring of intracranial pressure (ICP) in severe TBI patients. In the US, an average of about 20,586–25,634 (24,380) patients per annum undergo ventriculostomy (5).

Although TBI management is our main interest and the application of external ventricular drain (EVD) is a crucial point in TBI protocols, EVD infections are among the complications for EVD application with high influence on the outcome of the underlying disease and are not well-characterized.

Many conditions such as intracranial hemorrhage, intracranial tumor and hydrocephalus prompt the use of EVD. EVD application is frequently complicated by misplacement, hemorrhage, dislodgement, blockage, and most importantly infection (6). EVD infection rate ranges from 0 to 22% (7–9) resulting in a significant increase in cost, hospital stay, morbidity, and mortality (6, 10).

Due to the heterogeneous knowledge on the effectiveness of EVD, uncertainties of EVD application and the infection related complications, further research is required. The aim of this qualitative review was to identify risk factors that can potentially affect the incidence of EVD infection and create a model, which can be used in future studies to determine the real risk factors with their real strengths in order to contribute to the elaborations on the guideline for EVD application among TBI patients.

## Methods

We performed a systematic search on PubMed and Google Scholar databases (from 1966 to August 2017) for relevant studies related to ventricular drain infections. Keywords used in the search strategy include:

1. Infections (ventricular drain, ventriculostomy related, external ventricular drain, ventricular catheter, and extra-ventricular drain) and
2. One of the following [traumatic brain injury, Intensive care (ICU) patients', neuro-intensive care (NICU) patients, head injury, brain injury, cerebral hemorrhage, sub-arachnoid hemorrhage].

The combination of keywords generated 328 and 276 references on PubMed and Google Scholar, respectively. Of 604 references, 28 were found relevant after the title and abstract screening. In addition to these, the references of these 28 relevant articles were searched manually to find more related articles, which generated 4 new articles.

The 32 relevant articles were screened; those that performed a *multivariate analysis* of suspected risk factors (making a difference between factors which proved to be a confounding factor in a study and factors which can have an influence on outcome according to the published reports) and had a positive culture as a mandatory component in diagnosis were selected for data collection and analysis. Twenty articles (16 of these are as a result of the keyword-based literature search and 4 are as a result of manual reference search) were finally selected for our review. The cumulative sample size of the 20 studies was 5,113 patients, with a median of 164.5.

In our review, quantitative methods (meta-analysis) could not be applied due to the heterogeneity of the studies in respect to the risk factors identified i.e., the number of studies identifying a certain risk factor was small, making meta-analysis impossible.

As a result of the heterogeneity of the clinical terms used by the authors a common term was chosen for the varying terminologies. “Age” and “younger age” were simplified to ***age***; “co-infection,” “concurrent systemic infection,” “open infection source,” and “skin colonization by pathogen” were simplified to ***co-infection***; “Depressed Cranial Fracture repairing surgery (DSF),” “neurosurgical operation,” “length of tunneling (> 5cm),” “craniotomy,” and “two or more burr-holes” were simplified to ***neurosurgical operation***; “duration of catheterization,” “duration of catheterization (>11 days)” and “intracranial pressure monitoring (ICPM) > 5 days” were simplified to ***duration of catheterization***; “standard catheter,” “conventional catheter” and “antibiotic coated catheter” were simplified to ***catheter type***; “neuro-trauma,” “multiple trauma,” “sub-arachnoid hemorrhage (SAH),” “intraventricular hemorrhage (IVH),” “intracerebral hemorrhage (ICH),” and “intraventricular hemorrhage (IVH)” were simplified to ***diagnosis***; “repeat insertion,” “patients with >1 ICPM,” “multiple catheter replacements,” and “number of catheters” were simplified to ***multiple catheters***.

Duration of catheterization refers to the time period between post-catheter insertion and the detection of infection or discharge from ICU in the absence of infection.

According to CDC, meningitis or ventriculitis must meet at least one of the following criteria:

1. Patient has organism(s) identified from cerebrospinal fluid (CSF) by a culture or non-culture based microbiologic testing method which is performed for purposes of clinical diagnosis or treatment for example, not Active Surveillance Culture/Testing (ASC/AST).
2. Patient has at least two of the following:
  - fever (>38.0°C) or headache (Note: Elements of “i” alone may not be used to meet the two required elements)
  - meningeal sign(s)^*^
  - cranial nerve sign(s)^*^
3. Patient ≤1 year of age has at least two of the following elements: i. fever (>38.0°C), hypothermia, apnea^*^, bradycardia^*^, or irritability^*^ (Note: Elements of “i” alone may not be used to meet the required two elements).
  ii. meningeal signs^*^
  iii. cranial nerve signs^*^

^*^ With no other recognized cause

**And at least one of the following for Number 2 and two of the following for Number 3 listed above:**

- increased white cells, elevated protein, and decreased glucose in CSF (per reporting laboratory's reference range)
- organism(s) seen on Gram stain of CSF
- organism(s) identified from blood by a culture or non-culture based microbiologic testing method which is performed for purposes of clinical diagnosis or treatment, for example, not Active Surveillance Culture/Testing (ASC/AST)
- diagnostic single antibody titer (IgM) or 4-fold increase in paired sera (IgG) for organism

The definition of infection varied among authors. Some defined infection as a positive CSF culture from either the ventricular catheter or lumbar subarachnoid space, verified by growth on agar plates (11–13). Others defined infection by the CDC criteria for meningitis/ventriculitis (14, 15). Some authors defined infection with a few criteria, e.g., Mayhall et al. defined infection as; no other detectable source of central nervous system (CNS) infection, negative cultures of CSF obtained at the time of catheter insertion, ventricular catheterization for 24 h or longer and a positive CSF culture from either the ventricular catheter or lumbar subarachnoid space. Pople et al. also defined infection by a few criteria which includes; CSF culture with no organisms identified on initial Gram stain that subsequently grew a positive culture on agar, or CSF culture negative, but with Gram stain showing either gram-positive or gram-negative organisms, or CSF leukocytosis with a white blood cell/ red blood cell CSF count >0.02. Omar et al. defined infection as a positive CSF culture and Gram stain and presence of other supportive CSF laboratory findings such as pleocytosis with microscopic examination showing presence of white blood cells of more than 11/mm^3^, a decrease in the CSF glucose level and an increase in the CSF protein level. Standard catheter refers to catheters without hydrogel or silver or antibiotic(s) coating or impregnation while antibiotic coated catheter refers to catheters coated with antibiotic(s).

## Results

Table 1 summarizes the factors that proved to be significant or non-significant in the univariate analyses of the studies included in this review. Out of the 20 articles selected for analysis, three studies reported no significant association between the risk factors evaluated and EVD infection (6, 15, 17) after multivariate analysis. Altogether 15 risk factors (10 patient-related and 5 catheter-related) were identified by our review. Risk factors found by most investigations were neurosurgical operation and duration of catheterization. In general, the reviewed studies dealt only with a narrow set of possible risk factors. The maximum number of risk factors identified by a study was 5 (Table 2).

**Table 1:** Variables identified by univariate analysis having no significant association with EVD infection and factors which showed significant association with EVD infection only in univariate analyses.

**Study**	**Significant variables identified in uni-variate analyses**	**Non-significant variables identified in uni-variate analyses**
Arabi et al. (16)	Prophylactic antibiotics.	APACHE II; SAPS II; Placement of EVD outside the OR; ICU–LOS; Hospital LOS and Mortality.
Bari et al. (17)	–	–
Bota et al. (7)	–	APACHE II; LOS; ICU mortality rate and In-hospital mortality rate.
Camacho et al. (14)	Hospital–LOS; ASA I and Antibiotic prophylaxis.	Duration of surgery and Mortality.
Flibotte et al. (11)	Hospital – LOS and NICU - LOS	Admission GCS ≤ 9; In-hospital mortality and VP shunt.
Gozal et al. (18)	–	–
Hagel et al. (6)	ICU–LOS and Hospital LOS.	BMI; ASA; Accommodation and In-hospital mortality.
Hoefnagel et al. (12)	–	Operating time and Prophylactic antibiotic.
Holloway et al. (19)	–	–
Kirmani et al. (20)	Intraventricular antibiotic.	Steroid use.
Lo et al. (21)	–	Presence of trauma.
Mayhall et al. (22)	–	Underlying disease; Placement of EVD in ICU; Antibiotic prophylaxis; CSF drain Disconnections; Previous ventriculostomy and Other CNS instrument.
Mounier et al. (23)	–	Immunodeficiency; Recent neurosurgery; Antibiotics prophylaxis during EVD placement; Antibiotics administration during EVD drainage; EVD placement by resident; Emergency EVD placement; EVD exchange; Drainage lock and EVD disconnection.
Omar et al. (24)	–	Venue of surgery and Surgeon's status.
Paramore et al. (13)	–	Location of catheterization within the hospital.
Park et al. (25)	–	–
Peter et al. (10)	–	–
Pople et al. (26)	–	–
Rebuck et al. (15)	–	Skull fracture; Presence of multiple trauma; Penetrating head injury; Antibiotic prophylaxis and Location of catheter placement within the hospital
Wright et al. (27)	–	–

*EVD, External Ventricular drain; LOS, length of stay; (N)ICU, (Neuro) Intensive Care Unit; GCS, Glasgow Coma Scale; ASA, American Society of Anaesthesiologists; APACHE, Acute Physiology And Chronic Health Evaluation; SAPS, Simplified Acute Physiology Score; CSF, Cerebrospinal Fluid; CNS, Central Nervous System; ICPM, Intracranial Pressure Monitor; OR, Operating Room; BMI, Body Mass Index*.

**Table 2:** Identified risk factors of extra-ventricular drain infections according to the published results from 1984.

**Risk Factors**	**Arabi et al. (16)**	**Bari et al. (17)**	**Bota et al. (7)**	**Camacho et al. (14)**	**Flibotte et al. (11)**	**Gozal et al. (18)**	**Hagel et al. (6)**	**Hoefnagel et al. (12)**	**Holloway et al. (19)**	**Kirmani et al. (20)**	**Lo et al. (21)**	**Mayhall et al. (22)**	**Mounier et al. (23)**	**Omar et al. (24)**	**Paramore et al. (13)**	**Park et al. (25)**	**Peter et al. (10)**	**Pople et al. (26)**	**Rebuck et al. (15)**	**Wright et al. (27)**	**Number of positive publications**
Patient factors/Sample size	84	256	638	119	311	498	218	228	584	130	199	172	101	87	161	595	100	434	215	144	
Sex		NS	NS	NS	NS		NS			NS	X			NS		NS				NS	1
Age		NS	NS		X		NS			NS						NS	NS	NS		X	2
Age & sex interaction																				X	1
Co-infection			X						X	X			X						NS		4
CSF leakage										NS			X			NS			NS		1
Neurosurgical operation			X				NS		X	X		X		X			X				6
CSF sampling frequency								X													1
ICP > 20mmHg												X							NS		1
Diagnosis			X		NS		NS		X			X				NS	NS	NS			3
Reduced CSF glucose						X															1
Catheter factors																					
Multiple catheters	X	NS							NS		X						X		NS		3
Catheter insertion outside the hospital	NS															X					1
Duration of catheterization	NS			X	X		NS	X			NS	X	NS	X	X		X	X	NS		8
Catheter type							NS											NS	NS	X	1
Irrigation												X									1
Number of identified risk factors	1	_	3	1	2	1	_	2	3	2	2	5	2	2	1	1	2	1	_	3	

*X, factors investigated and found to be significant; NS, factors investigated but found to be non-significant*.

### Patient Factors

Age was identified by Flibotte et al. [OR & 95%CI: 1.04, 1.01–0.081; *P*-value: 0.03] and Wright et al. [HR & 95%CI: 1.051, (1.01–1.09) *P*-value, 0.014] as a risk factor associated with ventricular catheter infection. Both studies measured age in years and found a 4–5.1% increase in risk of EVD infection per annum respectively (11, 27).

Lo et al. reported that females [OR & 95%CI: 3.4, (1.2–9.7); *P*-value, 0.02] were three times as likely to be infected as males (21).

Age & sex interaction [HR & 95%CI: 0.912, (0.85–0.98); *P*-value, 0.0112] was identified as a risk factor for EVD infection by Wright at al. They also reported that female patients were 6 times likely to have an EVD infection than male patients (23.7% vs. 3.1%, OR: 6.4, *p* < 0.003) (27).

Co-infection, a risk factor with a higher incidence among the patient factors was identified by Bota et al. [OR & 95%CI: 3.92, (0.66–7.84); *P*-value, 0.02], Holloway et al. [*P*-value, 0.001], Kirmani et al. [*P*-value, 0.002], and Mounier et al. [OR & 95%CI: 11.8, (2.5–56.8); *P*-value, 0.002] and was found to be significantly associated with ventricular catheter infection (7, 19, 20, 23). The EVD infection rates of 12, 20.7, 15, & 37.5% in patients who had a concurrent infection vs. 7, 8.6, 6 & 4.7% in patients who did not were reported respectively (7, 19, 20, 23).

Mounier et al. reported cerebrospinal fluid (CSF) leakage at the site of insertion [OR & 95%CI: 10, (2.4–41.2); *P*-value, 0.001] as a risk factor and that among the infected patients, most of the catheter infection was as result of the colonization at the site of catheter insertion (23).

Diagnosis was identified by Bota et al. [(SAH: OR & 95%CI: 2.95, (0.59–5.26); *P*-value, 0.02), (IVH: OR & 95%CI: 2.07, (0.65–4.87) *P*-value, 0.02), (neurotrauma: *P*-value, 0.03)], Holloway et al. [*P*-value, 0.007], and Mayhall et al. [*P*-value, 0.027] as a risk factor with a high prevalence among the patient factors with significant influence on the incidence of ventricular catheter infection (7, 19, 22).

According to Hoefnagel et al. increased frequency of CSF sampling [OR & 95%CI: 4.12, (1.84–9.22); *P*-value, 0.001] is a risk factor to EVD infection. The authors noted that CSF was not always sampled according to the institution's protocol that had been set which inevitably increased the frequency of catheter manipulation and consequently the risk of infection (12).

According to Mayhall et al. intracranial pressure (ICP) above 20 mmHg [*P*-value, 0.019] is a risk factor for ventricular catheter infection but they mention also the alternate explanation for their observation, that patients with high ICP may need ventricular catheter for longer periods which predisposes them to infection (22).

Neurosurgical operation was identified by Bota et al. [OR & 95%CI: 4.74, (0.27–9.52); *P*-value, 0.03], Holloway et al. [craniotomy: *P*-value, 0.005; DSF: *P*-value, 0.003], Mayhall et al. [*P*-value, 0.016], Omar et al. [OR & 95%CI: 10.46, (3.38–32.32); *P*-value, 0.001], and Peter et al. [*P*-value, 0.047] as a risk factor (7, 10, 19, 22, 24). The reported infection rate varied between 15.2 and 82% in patients who underwent neurosurgical operation against 3.4 and 69% in patients who did not (7, 10, 19, 22, 24). Some authors reported that patients with one or more neurosurgical procedures were at a significantly higher risk for infection which may be due to immunosuppression or trauma associated with surgical procedures (20, 22).

Gozal et al. reported that there is a significant correlation between CSF glucose levels [OR & 95% CI: 4.87, (1.26–18.75); *P*-value, 0.021] drawn immediately after EVD placement associating <50% of serum glucose and subsequent risk of infection. Gozal et al. pointed out that this should not be mistaken for a pre-existing systemic infection since similar association was not found in CSF pleocytosis or protein levels which would have been expected in an on-going infection (18).

### Catheter Factors

Wright et al. reported catheter type [standard catheter vs. antibiotic coated catheter: HR & 95% CI: 0.091, (0.02–0.41); *P*-value, 0.007] as a risk factor associated with ventricular catheter infection. In the study carried out by Wright et al. two types of catheters (standard and antibiotic coated) were used. They reported infection rate as 23.5% for standard catheters and as 4.3% for antibiotic coated catheters. This represents a risk ratio of 0.18 and an absolute risk reduction of 19.2% after changing from standard catheter to antibiotic coated catheter (27).

Park et al. reported that patients that underwent catheter insertion outside the study center had a higher risk [HR & 95%CI: 3.42, (1.46–8.02); *P*-value, 0.005] of infection than patient that underwent catheter insertion in the study center and that there was limited information on the technique of catheter placement or specific location of the patient outside the study center at the time of catheter insertion. On the other hand, Park et al. also reported that the location of catheter insertion (OR/ICU/ED) within the study center did not significantly influence the infection rate of patient with catheters inserted in the study center (25).

Duration of catheterization was identified by Camacho et al. [OR & 95%CI: 1.08, (1.1–1.2); *P*-value, 0.036], Flibotte et al. [OR & 95%CI: 1.2, (1.1–1.3); *P*-value, 0.001], Hoefnagel et al. [OR & 95%CI: 4.12, (1.84–9.22); *P*-value, 0.001], Mayhall et al. [*P*-value, 0.017], Omar et al. [OR & 95%CI: 3.61, (1.19–10.94); *P*-value, 0.024], Paramore et al. [*P*-value, 0.016], Peter et al. [*P*-value < 0.001], and Pople et al. [RC & 95%CI: −0.048, (−0.092 to −0.003)] as a risk factor (10–14, 22, 24, 26). The duration of catheterization ranged from 1 to 44 days in these studies. Some authors suggest that the longer duration of catheterization may cause microbial infection of the catheter (13, 19, 22) while there is another opinion that the longer duration of catheterization is a consequence of the catheter infection rather than the cause (6).

Multiple catheters was identified Arabi et al. [OR & 95%CI: 6.34, (1.36–29.64); *P*-value, 0.019], Lo et al. [OR & 95%CI: 4.6, (2.3–9.2); *P*-value, < 0.0001], and Peter et al. [*P*-value, < 0.01] as a risk factor for EVD infection (10, 16, 21). Arabi et al. and Peter et al. reported an infection rate of 42 and 84.5% in patients who received multiple catheters vs. 3 and 18.3% in patients who did not respectively. Lo et al. also reported that infected patients used almost twice the amount of ventricular catheters as their uninfected counterparts (10, 16, 21). Each additional catheter was reported to increase the risk of infection by 4-fold or 8% by some authors (8, 21). Arabi et al. found out that antibiotics were given more frequently with first insertion than with repeated insertions [68 vs. 7%, *P*-value, 0.001] and it may be responsible for making multiple catheters a risk factor (16).

Mayhall et al. who identified irrigation [*P*-value, 0.021] as a risk factor for EVD infection also hypothesized that infections are likely introduced into the ventricles by retrograde movement of microbes due the manipulation of the catheter system (22).

## Discussion

The keywords and references used in our review were similar to the keywords and references in other reviews (8, 9, 28). Despite the sparse amount of articles on this topic, we were able to identify a total of 15 risk factors.

According to our review, distinct studies were able to identify only few risk factors, since the published studies were intentionally focused to a few specific causes of EVD infection with controlling for only few confounding factors, instead of testing a comprehensive set of causal factors representing the hypotheses on the etiological background. On the other hand, the sample size of reviewed studies was rather limited, preventing the effective identification of less dominant risk factors. Further, the terms of investigated clinical factors were highly variable, limiting the effectiveness of comparative evaluation. Therefore, the research on the risk factors of EVD infection is in its initial phase. Identification of factors which can play role in development of infective complications of EVD was the function of the till-now-published papers. The reviewed investigations could contribute to the building of research model to be tested in future, and cannot be considered as reliable quantification of the risk factors' role.

Although, only papers with multivariate models were analyzed in this review, as shown in Table 2, none of them could cover the majority of risk factors. Even the paper with the most extended model could not cover half of the identified risk factors. Consequently, the published measures of associations reflect both the strength of the risk factors and the confounding effects of factors that were not included in the model but were associated with the risk factors included in the studied models. Therefore, the relative importance of a risk factor cannot be evaluated by the published models. The whole set of suspected risk factors need to be included in the model which will be tested in clinical practice in order to determine the risk factors with their strength and clinical importance.

There were studies that found some of the identified risk factors not significantly associated with EVD infection in their multivariate analysis. Age, sex, CSF leakage, catheter type, and diagnosis were identified by more studies as not significantly associated with EVD infection (6, 7, 10, 11, 14, 15, 17, 20, 24–27) than studies that did. On the other hand, while more studies reported some risk factors to be significantly associated with EVD infection, a few studies did not find a similar association between these risk factors and EVD infection after multivariate analysis. Duration of catheterization, co-infection, and neurosurgical operation were found by more studies to be associated with EVD infection than a few studies that did not (7, 10–14, 16, 19–24, 26). Catheter insertion outside the hospital, multiple catheters, and ICP > 20 mmHg were found to be either associated with EVD infection or not by equal number of studies (15–17, 25–27). The varying results from the reviewed studies are possibly due to non-standardized research procedures (i.e., some risk factors were selectively analyzed while some risk factors were omitted, resulting in a possible causal or coincidental relationship or the lack thereof between EVD infection and these risk factors). Some of the factors presented in this review for later research may only be confounding factors without direct influence on ventricular catheter infection occurrence (e.g., the association between gender and drain infection; far more males were involved in severe injuries whereas females appeared to be a predictive factor).

This review has a few limitations. Firstly, not all the possible research papers were collected as only the PubMed and Google Scholar databases were used, also the research papers published in other languages other than English language were not included in this review. Secondly, as mentioned previously, the meta-analysis of the risk factors could not be carried out. Thirdly, some of the selected papers were not focused on EVD exclusively (some included ICP monitors). Lastly, the explanatory power of the proposed study model could not be determined because the studies evaluated only a narrow set of influencing factors. Consequently, the proposed study model may include interrelated prognostic factors and it is not possible to predict whether factors omitted from our proposed research model (risk factors) have high impact on manifestation of EVD infection.

Application of ICP monitoring has recently become a major topic of discussions in the scientific community also leading to the conduct of major randomized clinical trial (BEST-TRIP) (29). Nevertheless, to much of our surprise, application of such a common monitoring and therapeutic tool is based on very limited knowledge of purported risk factors associated with its' utilization.

Being aware of such complications and their rate would be of ample importance to inform the relatives, train the care givers as well as enhance therapeutic efficacy. We hope that the present work not only focused attention at our above-detailed weaknesses but also highlighted those potential factors that should be considered when EVD-related infective complications are to be predicted.

## Conclusion

Studies published on risk factors of EVD infection till 2017 have serious limitations and can be considered only as preliminary investigations which yielded a set of variables (patients and EVD related factors) that should be covered by future observational epidemiological investigations. Despite the huge cumulative sample size 5,113 patients of these studies, the comparability of the results was seriously hampered by the non-standard assessment of investigated clinical factors. The outcome of our review is a recommendation that former approaches should be replaced by a design able to determine the clinical importance of factors related to EVD infection and able to prepare a formal quantitative meta-analysis. According to our results, the set of the parameters in the study model should be used at a minimum–besides other factors depending on the tested hypothesis in the etiology of EVD infection. These variables are: age, sex, age & sex interactions, co-infection, catheter insertion outside the hospital, catheter type, CSF leakage, CSF sampling frequency, diagnosis, duration of catheterization, ICP > 20 mmHg, irrigation, multiple catheter, neurosurgical operation, and reduced CSF glucose at insertion of ventricular catheter.

## Author Contributions

AS was responsible for the search of articles. JS was responsible for the structure of the content in the manuscript. AS, JS, and AB were responsible for the writing of the manuscript. AB, JS, and EC were responsible for the editing, proof-reading and finalization of the manuscript

### Conflict of Interest Statement

The authors declare that the research was conducted in the absence of any commercial or financial relationships that could be construed as a potential conflict of interest.
